# Supplementary material for: Generating context-specific sports training plans by combining generative adversarial networks
Source: PLoS One. 2025 Jan 30;20(1):e0318321. doi: 10.1371/journal.pone.0318321 (PMC11781690; doi:10.1371/journal.pone.0318321)
Supplement: Attachment — Submitted filename: PONE-D-24-34881_reviewer.pdf [file pone.0318321.s001.pdf]

PLOS ONE

Generating Personalized Sports Training Plans by Combining Generative Adversarial Networks (GAN)

--Manuscript Draft--

**Manuscript Number:**	PONE-D-24-34881
**Article Type:**	Research Article
**Full Title:**	Generating Personalized Sports Training Plans by Combining Generative Adversarial Networks (GAN)
**Short Title:**	GAN-Driven Sports Training Plans
**Corresponding Author:**	Jingwen Chen<br>Beijing Normal University<br>Zhuhai, CHINA
**Keywords:**	Generative Adversarial Network; Personalized Sports Training Program; Deep neural networks; optimization algorithm; Adversarial Training
**Abstract:**	Currently, with the advancement of technology and the increasing intensity of sports competition, people are paying more and more attention to the rationality and effectiveness of sports training. However, traditional sports training programs are usually designed based on group data and average patterns, ignoring the differences and special needs of individual athletes, resulting in a lack of personalized, scientific, and targeted training programs, and unsatisfactory training results. To address this issue, this study combined Generative Adversarial Networks (GAN) technology to generate personalized motion training schemes to improve training effectiveness. Firstly, individual motion data and related features can be collected and preprocessed to ensure the quality and consistency of the dataset; secondly, a model architecture for GAN can be designed, including a generator and discriminator. The generator utilizes deep neural networks to generate personalized training plans, while the discriminator evaluates the authenticity of the generated plans. Next, loss functions and optimization strategies can be defined, appropriate loss functions can be selected to measure the performance of the generator and discriminator, and gradient descent algorithm can be used to iteratively optimize parameters; finally, through adversarial training, the quality and personalization of the generated plan can be continuously improved, thereby achieving personalized sports training plan generation. In order to evaluate the performance of combining GAN models to generate personalized training schemes in this article, comparative analysis experiments were conducted on the Sports Performance Dataset in UCI Machine Learning Repositories with traditional models such as machine learning models. In terms of generation speed, the average generation time of the GAN model was 15.7 minutes. The average generation time of traditional machine learning models was 36.4 minutes, which improved the efficiency of generating personalized motion training plans by about 56.9%. It also evaluated the personalization, scientificity, applicability, and feasibility of the proposed model and traditional model in generating solutions through a survey questionnaire. The results indicate that the model in this article has a higher evaluation in these aspects and obvious advantages. This reflects that the scheme generated by the model in this article is closer to the actual needs of individual athletes, more personalized and targeted, and can provide more personalized and effective sports training plans for athletes, improving their training effectiveness.
**Order of Authors:**	Juquan Tan
	Jingwen Chen
**Additional Information:**	
**Question**	**Response**
**Financial Disclosure**<br><br>Enter a financial disclosure statement that describes the sources of funding for the	The author(s) received no specific funding for this work.

Powered by Editorial Manager® and ProduXion Manager® from Aries Systems Corporation

work included in this submission. Review the submission guidelines for detailed requirements. View published research articles from *PLOS ONE* for specific examples.<br><br>This statement is required for submission and **will appear in the published article** if the submission is accepted. Please make sure it is accurate.<br><br>**Funded studies**<br>Enter a statement with the following details:<br>• Initials of the authors who received each award<br>• Grant numbers awarded to each author<br>• The full name of each funder<br>• URL of each funder website<br>• Did the sponsors or funders play any role in the study design, data collection and analysis, decision to publish, or preparation of the manuscript?<br><br>Did you receive funding for this work?	
**Competing Interests**<br><br>Use the instructions below to enter a competing interest statement for this submission. On behalf of all authors, disclose any competing interests that could be perceived to bias this work—acknowledging all financial support and any other relevant financial or non-financial competing interests.<br><br>This statement is required for submission and **will appear in the published article** if the submission is accepted. Please make sure it is accurate and that any funding sources listed in your Funding Information later in the submission form are also declared in your Financial Disclosure statement.<br><br>View published research articles from *PLOS ONE* for specific examples.	The authors have declared that no competing interests exist.

**NO authors have competing interests**<br><br>Enter: *The authors have declared that no competing interests exist.*<br><br>**Authors with competing interests**<br><br>Enter competing interest details beginning with this statement:<br><br>*I have read the journal's policy and the authors of this manuscript have the following competing interests: [insert competing interests here]*<br><br>* typeset	
**Ethics Statement**<br><br>Enter an ethics statement for this submission. This statement is required if the study involved:<br><br>• Human participants<br>• Human specimens or tissue<br>• Vertebrate animals or cephalopods<br>• Vertebrate embryos or tissues<br>• Field research<br><br>Write "N/A" if the submission does not require an ethics statement.<br><br>General guidance is provided below. Consult the submission guidelines for detailed instructions. **Make sure that all information entered here is included in the Methods section of the manuscript.**	This study was conducted in full accordance with the ethical standards of the South China Agricultural University's Institutional Review Board . Approval number: 20240401.

Powered by Editorial Manager® and ProduXion Manager® from Aries Systems Corporation

**Format for specific study types**<br><br>**Human Subject Research (involving human participants and/or tissue)**<br>• Give the name of the institutional review board or ethics committee that approved the study<br>• Include the approval number and/or a statement indicating approval of this research<br>• Indicate the form of consent obtained (written/oral) or the reason that consent was not obtained (e.g. the data were analyzed anonymously)<br><br>**Animal Research (involving vertebrate animals, embryos or tissues)**<br>• Provide the name of the Institutional Animal Care and Use Committee (IACUC) or other relevant ethics board that reviewed the study protocol, and indicate whether they approved this research or granted a formal waiver of ethical approval<br>• Include an approval number if one was obtained<br>• If the study involved *non-human primates*, add *additional details* about animal welfare and steps taken to ameliorate suffering<br>• If anesthesia, euthanasia, or any kind of animal sacrifice is part of the study, include briefly which substances and/or methods were applied<br><br>**Field Research**<br><br>Include the following details if this study involves the collection of plant, animal, or other materials from a natural setting:<br>• Field permit number<br>• Name of the institution or relevant body that granted permission	
**Data Availability**<br><br>Authors are required to make all data underlying the findings described fully available, without restriction, and from the time of publication. PLOS allows rare exceptions to address legal and ethical concerns. See the PLOS Data Policy and FAQ for detailed information.	Yes - all data are fully available without restriction

A Data Availability Statement describing where the data can be found is required at submission. Your answers to this question constitute the Data Availability Statement and **will be published in the article**, if accepted.<br><br>**Important:** Stating 'data available on request from the author' is not sufficient. If your data are only available upon request, select 'No' for the first question and explain your exceptional situation in the text box.<br><br>Do the authors confirm that all data underlying the findings described in their manuscript are fully available without restriction?	
**Describe where the data may be found in full sentences. If you are copying our sample text, replace any instances of XXX with the appropriate details.**<br><br>• If the data are **held or will be held in a public repository**, include URLs, accession numbers or DOIs. If this information will only be available after acceptance, indicate this by ticking the box below. For example: *All XXX files are available from the XXX database (accession number(s) XXX, XXX.)*.<br>• If the data are all contained **within the manuscript and/or Supporting Information files**, enter the following: *All relevant data are within the manuscript and its Supporting Information files.*<br>• If neither of these applies but you are able to provide **details of access elsewhere**, with or without limitations, please do so. For example:<br><br>*Data cannot be shared publicly because of [XXX]. Data are available from the XXX Institutional Data Access / Ethics Committee (contact via XXX) for researchers who meet the criteria for access to confidential data.*<br><br>*The data underlying the results presented in the study are available from (include the name of the third party*	All relevant data are within the manuscript.

and contact information or URL).<br>• This text is appropriate if the data are owned by a third party and authors do not have permission to share the data.<br><br>* typeset	
Additional data availability information:	

Manuscript Click here to access/download;Manuscript;Manuscript.docx

Generating Personalized Sports Training Plans by Combining Generative Adversarial Networks (GAN)

Juquan Tan[1,a] , Jingwen Chen[2,b*]
[1]College of P.E.Teaching,South China Agricultural University, 510100 Guangzhou Guangdong,China
[2]College of Education for the Future,Beijing Normal University,519085 Zhuhai Guangdong,China
[a]tanjuquan@scau.edu.cn
[b]jingwen@bnu.edu.cn
[*]Corresponding Author

Abstract: Currently, with the advancement of technology and the increasing intensity of sports competition, people are paying more and more attention to the rationality and effectiveness of sports training. However, traditional sports training programs are usually designed based on group data and average patterns, ignoring the differences and special needs of individual athletes, resulting in a lack of personalized, scientific, and targeted training programs, and unsatisfactory training results. To address this issue, this study combined Generative Adversarial Networks (GAN) technology to generate personalized motion training schemes to improve training effectiveness. Firstly, individual motion data and related features can be collected and preprocessed to ensure the quality and consistency of the dataset; secondly, a model architecture for GAN can be designed, including a generator and discriminator. The generator utilizes deep neural networks to generate personalized training plans, while the discriminator evaluates the authenticity of the generated plans. Next, loss functions and optimization strategies can be defined, appropriate loss functions can be selected to measure the performance of the generator and discriminator, and gradient descent algorithm can be used to iteratively optimize parameters; finally, through adversarial training, the quality and personalization of the generated plan can be continuously improved, thereby achieving personalized sports training plan generation. In order to evaluate the performance of combining GAN models to generate personalized training schemes in this article, comparative analysis experiments were conducted on the Sports Performance Dataset in UCI Machine Learning Repositories with traditional models such as machine learning models. In terms of generation speed, the average generation time of the GAN model was 15.7 minutes. The average generation time of traditional machine learning models was 36.4 minutes, which improved the efficiency of generating personalized motion training plans by about 56.9%. It also evaluated the personalization, scientificity, applicability, and feasibility of the proposed model and traditional model in generating solutions through a survey questionnaire. The results indicate that the model in this article has a higher evaluation in these aspects and obvious advantages. This reflects that the scheme generated by the model in this article is closer to the actual needs of individual athletes, more personalized and targeted, and can provide more personalized and effective sports training plans for athletes, improving their training effectiveness.

Keywords: Generative Adversarial Network; Personalized Sports Training Program; Deep Neural Networks; Optimization Algorithm; Adversarial Training

1. Introduction

In the process of cultivating excellent athletes, sports training is a very important link [1]. With the rapid development of technology and the continuous improvement of sports competition level, enhancing the effectiveness of sports training through modern technology has become an important

means to improve the competitive level of athletes [2-3]. The different growth and development patterns of the human body result in differences among individuals at different stages of each project. Traditional sports training methods often rely on the coach's own experience to provide suggestions [4], lacking personalized [5-6], scientific [7-9], and targeted guidance for students [10]. This not only affects the training effectiveness of athletes, but may also lead to resource waste and physical injuries to athletes. Therefore, it is particularly important to explore a method that can generate personalized sports training plans. This article combines GAN technology to generate personalized exercise plans, providing athletes with more personalized and effective training plans to improve their training effectiveness and competitive level.

Through reviewing relevant literature, it has been found that in recent years, many scholars have explored personalized generation of sports training programs by using different techniques [11-12]. Shin D [13] used a large language model to develop personalized exercise plans. Although this method can assist in generating personalized training plans, it ignores the real-time feedback and dynamic changes of athletes, resulting in a lack of real-time and flexibility in the training plan. Cao X [14] used machine learning based image processing techniques to provide personalized training for football players. Although this method improves the personalization of the training plan to a certain extent, there are still problems such as weak model generalization ability and lack of diversity. Li Q [15] applied computer Internet of Things technology in gymnastics teaching and training, which can provide personalized training suggestions. The application of IoT technology requires corresponding equipment and infrastructure support, relying too heavily on specific devices and platforms.

In order to overcome the problems of the above methods, some researchers have attempted to apply GAN technology to generate personalized exercise training plans. GAN is a deep learning architecture [16-17]. Compared with traditional methods, GAN based technology has the ability to generate high-quality and diverse samples, as well as the advantages of real-time personalized scheme generation. A large number of researchers have also studied GAN technology in fields such as data generation and personalized recommendation. Wu Xiaoguang [18] used conditional GAN to solve the problem of incomplete characterization of personalized features in human gait generation. Cao X [19] trains personalized models for customers through GAN to better meet their personal needs. Wen J [20] generated a personalized recommendation framework based on conditional GAN. Yoon J [21] used the GAN framework to generate synthetic data, minimizing patient recognizability, to achieve more accurate decision-making and personalized treatment. Yuan C [22] applied GAN to personalized sentence generation, which combines commonly used function words and content words as input features of the generator, and generates personalized sentences by discriminating constraint conditions through a discriminator. Ali Z [23] utilized GAN to generate a recommendation model that helps users provide personalized citations. Shi Qian [24] applied Conditional Generative Adversarial Nets (CGAN) to personalized clothing recommendation and generation. Sun Jinyang [25] used GAN to provide users with personalized paper resources, generating resources that match user interests and preferences by using semantic features of papers as input features. Based on GAN, Chen Jiwei [26] models users' long-term stable preferences and recent dynamic preferences through a game of generator and discriminator, providing personalized recommendations for users. Gao Q [27] used an adversarial network consisting of a generator and discriminator to generate personalized travel suggestions, and demonstrated the effectiveness and efficiency of the proposed model through experiments. Through the research conducted by the above researchers on GAN technology, it has been found that GAN generates new data by simulating the distribution of real data, which has strong generation ability and

flexibility. Therefore, it is feasible to apply GAN to the personalized generation of sports training plans in this article, which can utilize the multidimensional information of athletes to generate personalized training plans and dynamically adjust them based on real-time feedback from athletes.

The purpose of this study is to use GAN to generate personalized sports training plans, in order to address the shortcomings of personalization and lack of scientificity in traditional training plans. By collecting and analyzing multidimensional information such as athlete physical fitness data and training performance, a GAN model suitable for sports training can be constructed. This model can be able to generate training plans that match the individual characteristics of athletes, and its effectiveness can be verified through comparative experiments. The research methods and results of this article can provide more scientific and personalized guidance for athlete training, and promote innovative development in the field of sports training.

2. Generation of Personalized Sports Training Plans on Basis of GAN

2.1 Data Collection and Preprocessing

The recruitment period for this study began on 01/04/2024 and ended on 01/05/2024.In the data collection stage, this article first collected individual athlete's sports training video data and personal feature data using the Olympic Sports Dataset dataset. Among them, personal characteristic data includes age, height, weight, heart rate, etc. Sports training video data requires a series of preprocessing operations such as video frame extraction, image preprocessing, and motion posture annotation to analyze information such as athlete's motion posture, intensity, and frequency during the training process.

2.1.1 Video Frame Extraction

When preprocessing motion training videos, the first step is to perform video frame extraction on the collected motion training videos, which converts the videos into continuous static images for subsequent image processing and analysis. Here, the image processing tool OpenCV can be used to extract frames from the video. Firstly, it can use the cv2. VideoCapture() function to open the video file and pass in the path of the video file. Then, the video. read() method can be used to loop through each frame of the video. For each frame of the image, it can be named using the frame counter frame_count and saved using the cv2. imwrite() function. Figure 1 shows the effect of partially extracting video frames.

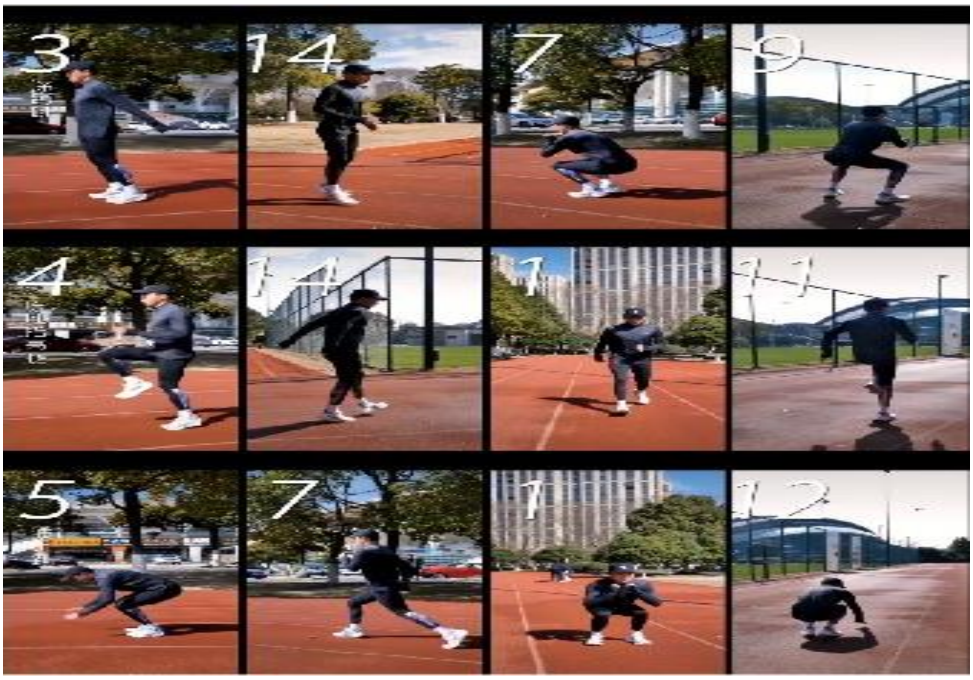

Figure 1 Video frame extraction effect

2.1.2 Image Preprocessing

After performing frame extraction on motion videos, the next step is to preprocess the video frame images, including image sharpening, contrast enhancement, and noise filtering. The purpose of these operations is to eliminate noise in the image, enhance the features of the image, and improve the quality and clarity of the image, providing more accurate image data for subsequent motion pose annotation. Firstly, the image can be sharpened to enhance its high-frequency details and make it clearer and more vivid. In this study, Laplacian filters can be used to achieve sharpening processing. Laplace filter is a commonly used edge detection filter that highlights high-frequency details in an image by performing second-order differentiation on the image [28]. The processing process is shown in formula 1.

$$Sharpened(x,y) = Input(x,y) + k*(Input(x,y) - Laplacian\_Filtered(x,y)) \quad (1)$$

In this formula, $Sharpened(x,y)$ represents the pixel value in the sharpened image, and $Input(x,y)$ is the original image value. k is the sharpening parameter, and $Laplacian\_Filtered(x,y)$ is the pixel value in the original image processed by a Laplace filter.

After the sharpening process is completed, it is necessary to perform contrast enhancement on the image. Contrast enhancement is achieved by adjusting the brightness distribution of the image to enhance its contrast. Histogram equalization is used here to achieve contrast enhancement. Histogram equalization expands the dynamic range of the image by reallocating pixel values by calculating the cumulative distribution function (CDF) of the image [29]. The implementation process is shown in formula 2.

$$Enhanced(x,y) = \frac{CDF(Input(x,y)) - \min\_CDF}{\max\_CDF - \min\_CDF} \times (\max\_pixel\_value - \min\_pixel\_value) + \min\_pixel\_value \quad (2)$$

Here, $Enhanced(x, y)$ represents the pixel value of the image after contrast enhancement, and $CDF(Input(x, y))$ represents the cumulative distribution function of the input image. $\min\_CDF$ and $\max\_CDF$ are the minimum and maximum cumulative distribution values of the input image, respectively. $\min\_pixel\_value$ and $\max\_pixel\_value$ are the minimum and maximum pixel values of the output image, respectively. After equalization through this histogram, the details and contrast in the image can be enhanced, making the image clearer and fuller. Finally, noise filtering can be applied to the image to reduce noise interference and improve the quality and clarity of the image. Here, noise filtering can be achieved using a Gaussian Filter, which is a linear smoothing filter that reduces noise by applying spatial Gaussian smoothing to the image [30]. It uses a Gaussian function to weighted average the pixels in the image, suppressing noise in the image. The processing process is shown in formula 3.

$$Filtered(x,y) = \sum_{i=-k}^{k} \sum_{j=-k}^{k} \frac{1}{2\pi\sigma^2} e^{-\frac{i^2+j^2}{2\sigma^2}} \times Input(x+i, y+i) \quad (3)$$

Among them, $Filtered(x, y)$ represents the pixel value of the image after noise filtering; σ is the standard deviation of the Gaussian kernel, and $Input(x+i, y+i)$ represents the pixel value of the input image at position $(x+i, y+i)$. After these image preprocessing operations, the image is shown in Figure 2.

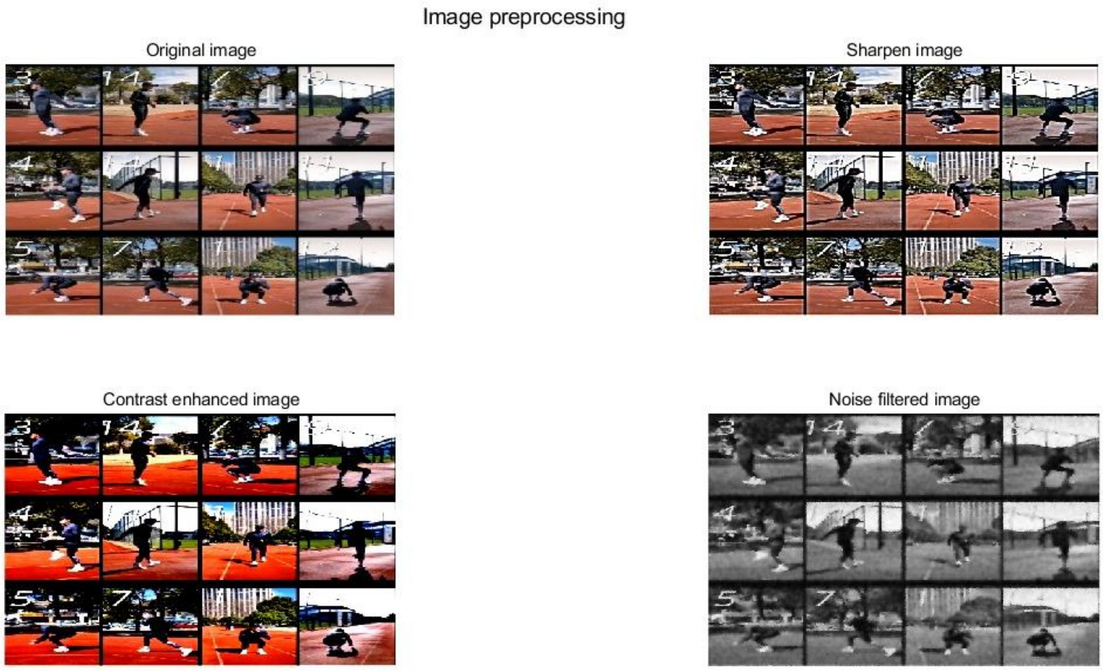

Figure 2 Image preprocessing effect

2.1.3 Sports Posture Annotation

In the stage of sports posture annotation, this article adopts a method based on human key point detection to locate key points in the preprocessed image, in order to annotate the athlete's posture information for subsequent analysis. This method automatically detects the key point positions of athletes in images through an OpenPose model based on deep learning. It uses a deep convolutional neural network to extract features from the image and predicts the position of key points through a regression network. The mathematical expression is shown in formula 4.

$$Heatmap(x,y,c) = \exp(-\frac{(x-x_c)^2+(y-y_c)^2}{2\sigma^2}) \quad (4)$$

Here, $Heatmap(x,y,c)$ represents the heatmap of key point positions; σ is the standard deviation of Gaussian kernel, and c is the category of key points. By training the model, accurate key point positions can be obtained, which in turn annotates the athlete's posture information. The results of motion posture annotation are shown in Figure 3. By annotating the postures of athletes in training videos, it can analyze the habitual posture and action information of athletes during the training process, preparing for personalized program recommendations in the future.

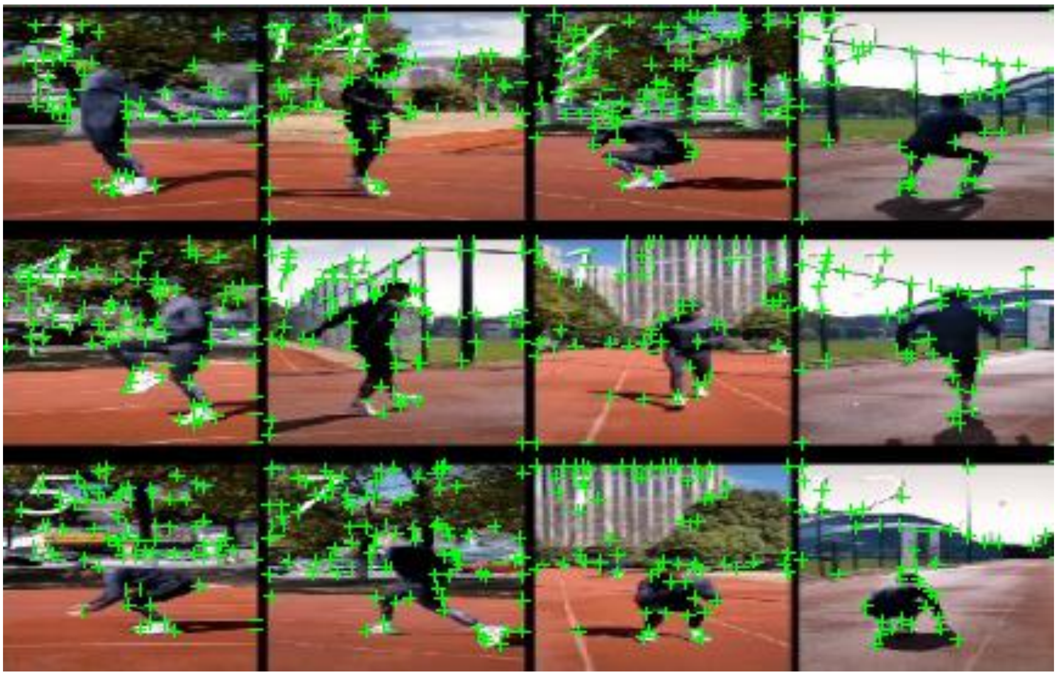

Figure 3 Sports posture annotation diagram

2.2 GAN Model Architecture Design

This article designs a GAN based model architecture to achieve personalized motion training scheme generation. Among them, the GAN system includes two deep neural networks - the generator network and the discriminator network [31-32]. These two networks train models in adversarial games. One of the networks attempted to generate new data, another network attempts to predict whether the output is fake or real data, so the model architecture consists of a generator and a discriminator. The task of the generator is to generate personalized training plans [33], and the task of the discriminator is to evaluate the authenticity of the generated plans [34-35]. The model architecture diagram is shown in Figure 4.

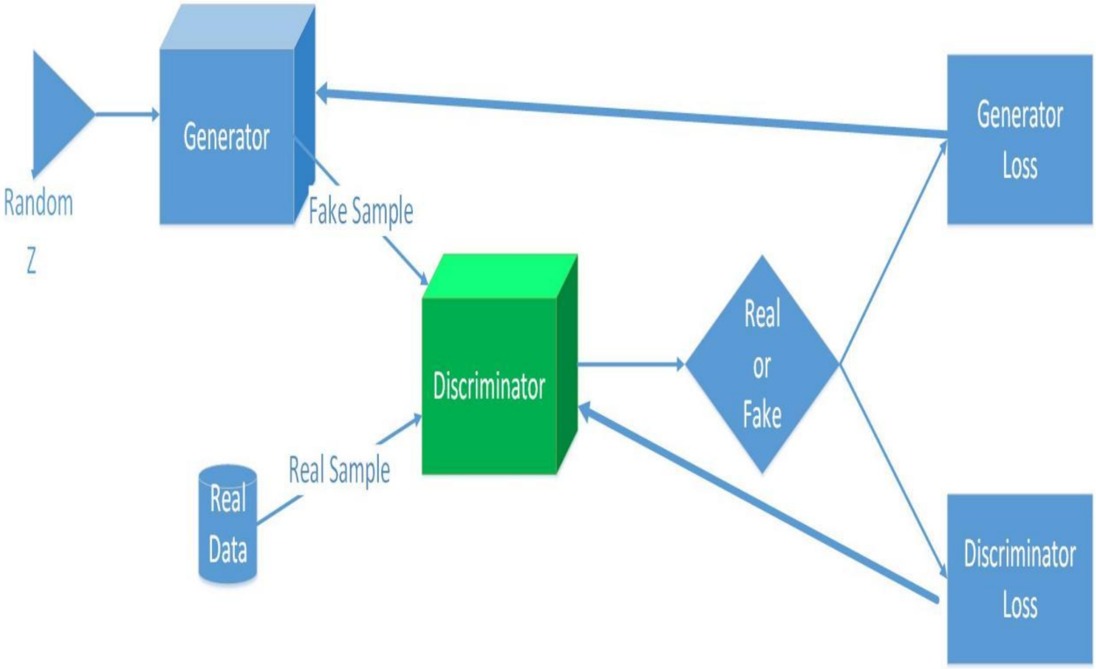

Figure 4 GAN model architecture diagram

The generator adopts a deep neural network (DNN) as its infrastructure. It consists of multiple hidden layers and activation functions, used to learn and generate personalized motion training schemes, and uses a multi-layer fully connected network to build the generator. The task of the generator is to map the input individual athlete's feature data x to the training scheme space, and output a personalized sports training scheme $G(x)$, whose mathematical expression is shown in formula 5.

$$G(x) = \mathrm{ReLU}(W_{out} \cdot \mathrm{ReLU}(W_{h2} \cdot \mathrm{ReLU}(W_{h1} \cdot x + b_{h1}) + b_{h2}) + b_{out}) \quad (5)$$

Among them, x is the input individual athlete feature data; W_{h1} and b_{h1} are the weights and biases of the first hidden layer; W_{h2} and b_{h2} are the weights and biases of the second hidden layer; W_{out} and b_{out} are the weights and biases of the output layer; and ReLU represents the modified linear unit function.

The discriminator, as another part of the GAN model, is another deep neural network used to evaluate the authenticity of the generated schemes. The discriminator receives the scheme generated by the generator and the real scheme as inputs, and outputs a probability value between 0 and 1, indicating the probability that the input scheme is the real scheme. Its mathematical expression is shown in formula 6.

$$D(x) = \sigma(W_d x + b_d) \quad (6)$$

Here, $D(x)$ represents the output discrimination result; σ is the activation function; W_d is the convolution kernel, and b_d is the bias term. By continuously alternating the training of the generator and discriminator, the generator is ultimately able to generate high-quality and personalized motion training plans, while the discriminator can accurately distinguish between real samples and generated samples.

2.3 Defining Loss Functions and Optimization Strategies

In this study, in order for GAN to effectively generate personalized motion training plans, it is necessary to define appropriate loss functions and optimization strategies. The loss function is used to measure the performance of the generator and discriminator, while optimization strategies are used to iteratively update network parameters to minimize the loss function. Firstly, the loss function of the generator can be defined. In order to make the generated personalized training scheme as close as possible to the real scheme, the average log likelihood loss function was used as the loss function of the generator to measure the difference between the generated scheme and the real scheme. The implementation process is shown in formula 7.

$$L_G = -\frac{1}{N}\sum_{i=1}^{N}\log(D(G(x_i))) \quad (7)$$

In this formula, N represents the number of training samples; x_i represents the input individual motion data; $G(x_i)$ represents the personalized training plan generated by the generator

on the input data x_i, and $D()$ represents the discriminator. The goal of the generator is to minimize the loss function in order to improve the authenticity of the generated scheme. Next, the loss function of the discriminator can be defined, with the goal of accurately distinguishing between the generated scheme and the actual scheme. To achieve this goal, the cross entropy loss function can be used as the loss function of the discriminator, as represented by formula 8.

$$L_D = -\frac{1}{N}\sum_{i=1}^{N}(\log(D(x_i)) + \log(1 - D(G(x_i)))) \quad (8)$$

Here, $D(G(x_i))$ represents the evaluation result of the discriminator on the generation scheme $G(x_i)$. The goal of the discriminator is to minimize the loss function, enabling the discriminator to accurately distinguish between generated and real schemes, thereby driving the generator to generate more realistic personalized training schemes.

In terms of optimization strategy, the Stochastic Gradient Descent (SGD) algorithm can be used to iteratively update the parameters of the generator and discriminator to optimize the parameters in GAN. For the generator, it updates the generator parameters based on the negative gradient of the generator's loss function, and the implementation process is shown in formula 9.

$$\theta_G = \theta_G - \eta \cdot \nabla_{\theta_G} L_G \quad (9)$$

In this formula, θ_G represents the parameters of the generator; η is the learning rate, and $\nabla_{\theta_G} L_G$ is the gradient of the generator loss function with respect to the parameters. By using the backpropagation algorithm, gradients can be calculated and the learning rate can be used to control the update speed of parameters. For the discriminator, the parameters are updated based on the negative gradient of the loss function of the discriminator, as shown in formula 10.

$$\theta_D = \theta_D - \eta \cdot \nabla_{\theta_D} L_D \quad (10)$$

Here, θ_D represents the parameters of the discriminator, and $\nabla_{\theta_D} L_D$ is the gradient of the discriminator loss function with respect to the parameters. By defining the loss function above and implementing optimization strategies, the parameters of the generator and discriminator can be iteratively updated, gradually improving the quality and personalization of the generated plan, thereby achieving the generation of personalized motion training plans.

2.4 Conduct Adversarial Training

Adversarial training is the core part of GAN, aimed at improving the quality and personalization of generated training schemes through the game process between the generator and discriminator. In the process of generating personalized solutions through adversarial training, the preprocessed dataset is first used for model training. This includes inputting individual feature data into the generator, generating personalized motion training plans, and simultaneously inputting the generated and real plans into the discriminator for adversarial learning. In the adversarial training process, the goal is to alternate the maximum and minimum adversarial loss functions as shown in formula 11 to optimize the parameters of the generator and discriminator.

$$\min_G \max_D V(D,G) = \mathrm{IE}_{x \sim p_{data}(x)}[\log D(x)] + \mathrm{IE}_{z \sim p(z)}[\log(1 - D(G(z)))] \quad (11)$$

Among them, G represents the generator; D represents the discriminator; $\mathbb{E}$ is the expected value; $p_{date}(x)$ represents the distribution of real data; z represents the input noise vector of the generator, and $p(z)$ represents the noise distribution. Through this iterative adversarial training process, the generator and discriminator compete and adjust with each other, ultimately achieving a dynamic equilibrium. The generator generates personalized training plans that are closer to the real plan by deceiving the discriminator, while the discriminator improves its discriminative ability by accurately distinguishing between the generated plan and the real plan. In this way, the generated sports training plan can better meet the special needs of individual athletes, improve training effectiveness and sports performance level.

3. Model Evaluation and Result

3.1 Experimental Setup

(1) Experimental environment configuration

This experiment used a server equipped with NVIDIA GeForce RTX 3090 GPU, Intel Core i9-11900K CPU, and 32GB memory to implement the GAN model using Python programming language and deep learning framework TensorFlow.

(2) Dataset selection

In order to evaluate the personalized sports training scheme generated by GAN, this experiment selected the Sports Performance Dataset from UCI Machine Learning repository as the experimental dataset. This dataset contains a large amount of athlete's sports data and related features, which can be used to train and evaluate the model in this paper. In terms of data selection, data from 100 athletes can be selected as experimental samples from the dataset, and representative athletes are selected for the experiment based on their different characteristics and needs. This can ensure that the model is evaluated on different types of athletes to verify its effectiveness in generating personalized plans.

3.2 Experimental Process

In order to evaluate the performance of combining GAN to generate personalized motion training schemes, this experiment compares and analyzes the GAN model with three traditional algorithm models (machine learning based, rule-based, and statistical based). In terms of experimental setup, the dataset can be divided into training and testing sets, using 30% of the data as the training set and 70% as the testing set. Next, for each model, this experiment first objectively calculated the evaluation indicators such as generation speed, mean square error value, and response speed for each model when generating personalized solutions. In addition, it also conducted subjective evaluation comparisons and collected feedback from these 100 athletes on personalized plans generated by different methods. It can collect feedback from athletes on their preferences, feasibility, adaptability, and other aspects of the plan through a questionnaire survey. Through comprehensive analysis of experimental results and subjective evaluation comparison, the performance of GAN model and traditional algorithm model can be comprehensively evaluated and compared to draw conclusions.

3.3 Experimental Results

(1) Display of personalized sports training plans generated by combining GAN

Firstly, in order to verify the feasibility of combining GAN to generate personalized sports training plans in this article, a running athlete can be used as an example here. After inputting the

athlete's characteristic data into the GAN model, taking into account the runner's physical fitness, technical level, and training objectives, a personalized training plan suitable for this athlete is generated. The training plan includes daily training for a week, including physical fitness training, technical training, and specialized training, as well as rest time. An example of the generated plan is shown in Table 1.

Table 1 Example of personalized sports training program

Date	Training program	Duration (hours)	Strength
Monday	Aerobic running	1.5	Medium
	Core training	0.5	High
Tuesday	Interval training	1.0	High
	Stretching training	0.5	Low
Wednesday	Aerobic running	1.5	Medium
	Technical exercises	1.0	Medium
Thursday	Interval training	1.0	High
	Core training	0.5	High
Friday	Aerobic running	1.5	Medium
	Stretching training	0.5	Low
Saturday	Long distance running	2.0	Medium
	Technical exercises	1.0	Medium
Sunday	Rest		

(2) The variation curve of the loss function during the training iteration process

In this experiment, the convergence of the GAN model was observed by plotting the changes in the loss function curve during the training process. The loss function curve includes two parts: generator loss and discriminator loss, where the generator loss reflects the performance of the generator network in generating personalized motion training schemes. The discriminator loss reflects the performance of the discriminator network in evaluating the authenticity of the generated scheme. This experiment records the unchanged loss function values of the generator and discriminator over 100 training iterations, as shown in Figure 5. Among them, the horizontal axis represents the number of training iterations, and the vertical axis represents the loss value. The blue curve represents the change in generator loss, while the red curve represents the change in discriminator loss. The lower the loss value of the generator, the closer the sample generated by the generator is to the real sample. The lower the loss value of the discriminator, the stronger its ability to distinguish between real and generated samples. As the number of training iterations increases, the loss value of the generator gradually decreases from around 0.8 and tends to stabilize. This indicates that the generator network gradually improves the quality of generated samples during the learning process, making them closer to real samples. The loss value of the discriminator gradually decreases from about 0.6 and fluctuates within a certain range, eventually stabilizing. This indicates that the discriminator network has gradually improved its ability to distinguish between real and generated samples during the learning process, and can more accurately determine the source of samples. Overall, the GAN model can effectively generate high-quality motion training schemes through the game process between the generator and discriminator. The discriminator can accurately distinguish between generated samples and real samples, which provides strong support for the design and application of personalized sports training programs.

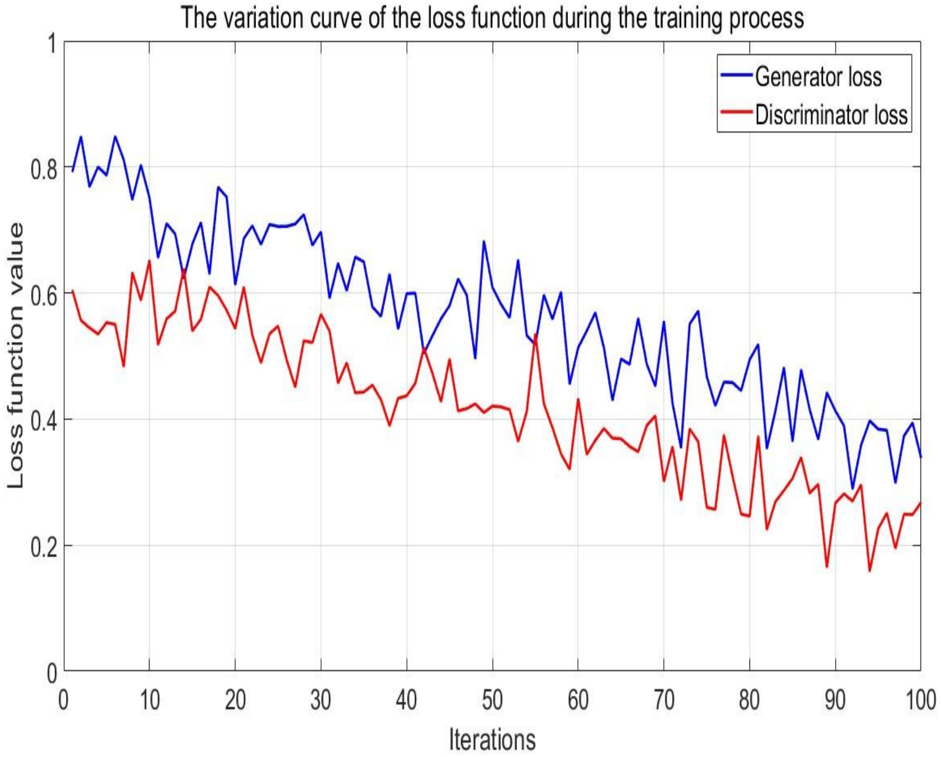

Figure 5 Loss function variation curve during training process

(3) Analysis of the Generation Speed of Personalized Training Plans

In order to evaluate the efficiency of combining GAN to generate personalized motion training schemes, this experiment compared and analyzed the GAN model (Model A) with three traditional algorithm models: machine learning based method (Model B), rule-based method (Model C), and statistical method (Model D). In this experiment, 10 athletes were randomly selected from the sample data, and the speed at which each model generated personalized sports training plans for each athlete was calculated. These data are presented in Table 2. The first column of Table 2 shows the athlete number, and the data in columns 2 to 4 represent the time required to generate personalized sports training plans for each athlete under the four models. From the data in Table 2, it can be roughly seen that each athlete has the shortest time to generate a plan under Model A (GAN model), which means the generation speed is the fastest.

Table 2 Record of Generation Speed for Each Model Scheme

Athlete number	Model A generation speed (minutes)	Model B generation speed (minutes)	Model C generation speed (minutes)	Model D generation speed (minutes)
1	15	35	40	38
2	14	33	38	36
3	16	37	42	40
4	15	35	39	37
5	17	39	41	39
6	16	37	40	38
7	18	41	43	41
8	14	33	38	36

9	19	43	44	42
10	13	31	37	35

In order to observe more intuitively the efficiency of the GAN model compared to traditional algorithm models in generating personalized sports training plans, the average generation time of personalized plans for these 10 athletes under each model was calculated here. The calculation results are shown in Figure 6. For these 10 athletes, the average generation time of the GAN model (Model A) studied in this article is the shortest of 15.7 minutes. The average generation time of traditional machine learning model (Model B) is 36.4 minutes; the average generation time of rule-based method (Model C) is 40.2 minutes; the average generation time of statistical method (Model D) is 38.2 minutes. The GAN model in this article has improved the efficiency of generating personalized motion training schemes by about 56.9% compared to traditional machine models. This indicates that the GAN model in this article has significant efficiency advantages in generating personalized sports training plans, and can generate personalized sports training plans for athletes faster and more effectively.

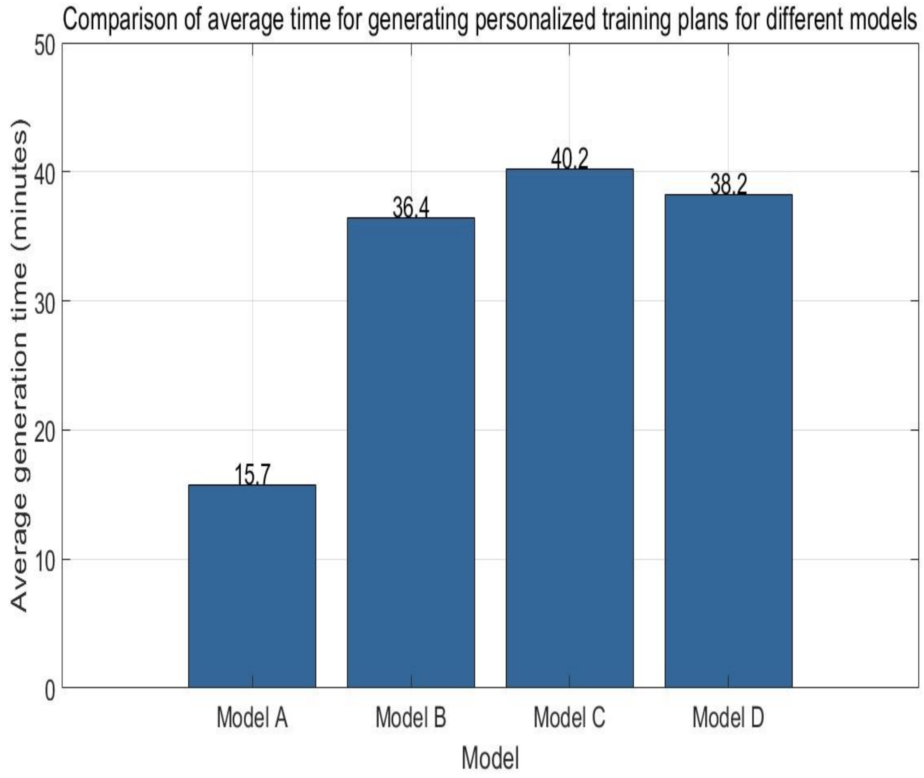

Figure 6 Comparison of average time for generating personalized training schemes for different models

(4) Comparative analysis of mean square error between the model in this article and traditional model generation schemes

Next, in order to explore the generation quality of personalized motion training schemes combined with GAN in this article, this experiment compared and analyzed the GAN model (Model A) with three traditional algorithm models: machine learning based method (Model B), rule-based method (Model C), and statistical method (Model D) on the mean squared error (MSE) index. Among them, mean square error is an indicator used to measure the average degree of difference between generated samples and real samples. A smaller mean square error value means that the difference between the generated personalized motion training scheme and the real sample is smaller, indicating that the generator performs better in terms of generation quality. In this experiment, 10 athletes were randomly selected

and their MSE values for generating personalized sports training plans under different models were calculated. The calculation results are shown in Figure 7. In Figure 7, the horizontal axis represents the athlete number and the vertical axis represents the MSE value. For these 10 athletes, the mean square error of the GAN model in generating personalized sports training plans fluctuates between 0.08 and 0.12 in this article. The mean square error values of the other three traditional algorithm models fluctuate between 0.13 and 0.22. Overall, the GAN model in this paper has a smaller mean square error value compared to other traditional models. This indicates that the combination of GAN model in this article can more accurately fit the needs and characteristics of athletes when generating personalized sports training plans. It can more effectively capture the feature distribution of samples and generate higher quality and more realistic personalized training plans.

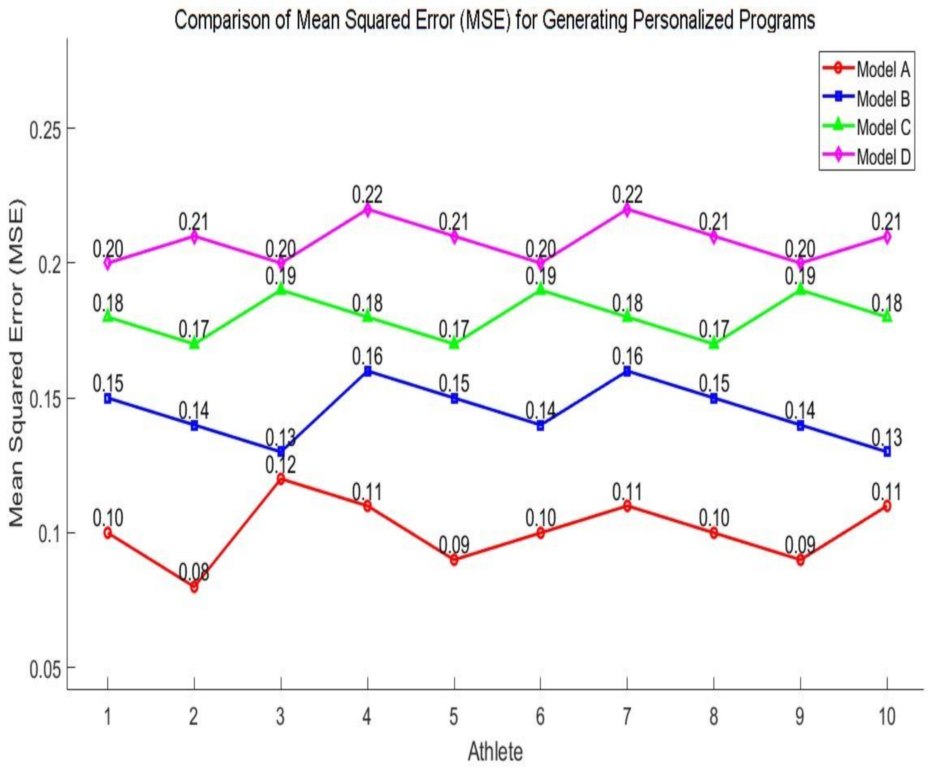

Figure 7 Comparison of mean square error for different models generating personalized schemes

(5) Comparative analysis of real-time performance between the model in this article and the traditional model in generating personalized solutions

In order to evaluate the real-time performance of combining GAN in generating personalized motion training schemes, this experiment compares the GAN model (Model A) with machine learning based methods (Model B), rule-based methods (Model C), and statistical methods (Model D) through response time indicators. Ten athletes were selected for the experiment, and the response speed during the process of generating personalized sports training plans for each athlete in each model was calculated. The calculation results are shown in Figure 8. From the data in Figure 8, it can be calculated that for these athletes, the average response time of the CAN model in generating personalized training plans in this article is 29.6ms. The average response times of traditional machine learning models, rule-based methods, and rule-based methods are 50.8ms, 64.8ms, and 58.2ms, respectively. These data indicate that the GAN model in this article has the lowest response time compared to other traditional models. This indicates that the GAN model has good real-time performance in generating personalized

training plans for athletes, providing real-time and efficient support for generating personalized sports training plans.

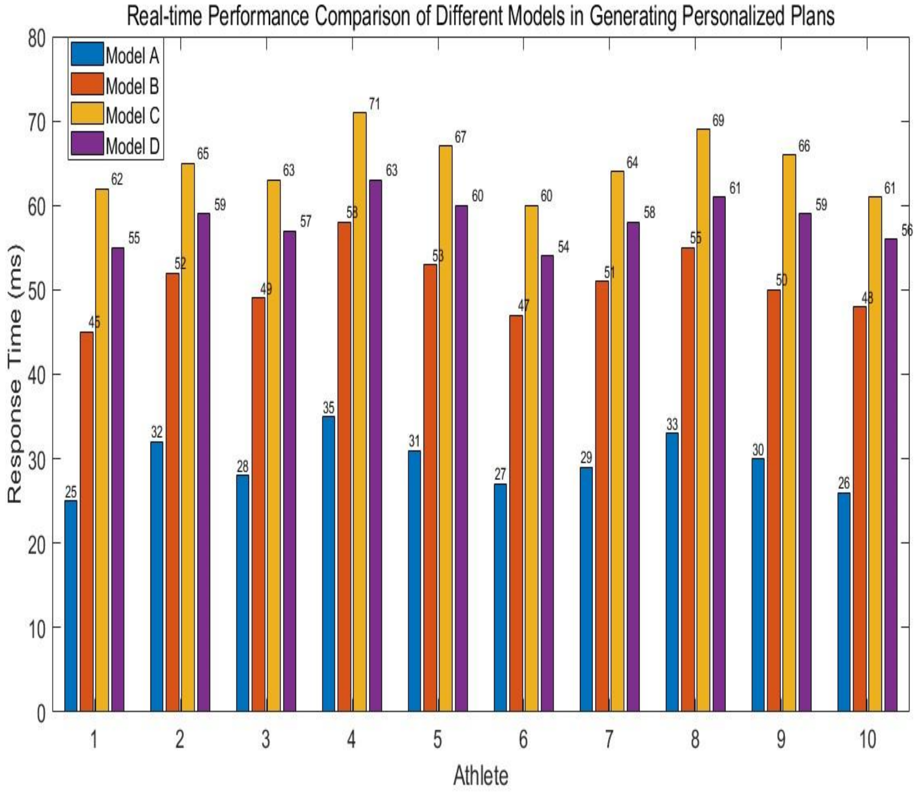

Figure 8 Real time comparison of personalized schemes generated by different models

(6) The subjective evaluation of personalized schemes generated by this article's model and traditional machine learning models

At the end of the experiment, a survey questionnaire was used to collect the evaluations of these 100 athletes on the personalized plans generated by the GAN model and traditional machine learning models. The evaluation content includes scoring the personalization, scientificity, applicability, and feasibility of the generated plan in four aspects, with a score of ten. This experiment collected and calculated the average scores of these 100 athletes, and recorded the results in Table 3. It can be seen that the GAN model in this article has achieved higher ratings compared to traditional machine learning models in four aspects of evaluation. This indicates that the personalized sports training plan generated by combining GAN is relatively reasonable. Its training plan that meets the needs of athletes themselves is more suitable for athletes to train and is conducive to improving the training effectiveness of athletes.

Table 3 Evaluation of personalized training schemes generated by different models

Model	Personalization Level	Scientificity	Applicability	Feasibility
GAN Model	9.32	9.00	8.57	8.80
Traditional Machine Learning Model	7.52	7.70	7.25	7.44

4. Conclusions

This article combined GAN technology to achieve personalized sports training plan generation for

athletes. The experimental research results indicate that by collecting individual motion data and features, and using deep neural networks to generate personalized plans, training effectiveness can be improved and the special needs of athletes can be met. Compared to traditional algorithm models, the scheme generated by the model in this paper has higher efficiency, quality, and real-time performance. However, there are also some shortcomings in this study, such as limitations in the data collection process, which may affect the data quality of the model, and the training process of the GAN model requires a large amount of computing resources and time. Future research can further optimize data collection methods to improve data quality and diversity, and explore more efficient training algorithms and model architectures to reduce training time and resource consumption. It can consider collaborating with domain experts to combine domain knowledge and model generated solutions to further improve the practical application effect of personalized training programs.

Statements & Declarations

Ethical Statement

This study was conducted in full accordance with the ethical standards of The Academic Committee of the Department of Physical Education Teaching and Research at South China Agricultural University. Approval number: 20240401.

Informed Consent

Participants have provided written informed consent and have been informed about the study's purpose, procedures, and data usage. They have been assured that participation is voluntary, they can withdraw at any time, and no personal information will be disclosed.

Author Contributions

Conceptualization, J.T.; methodology, J.C.; software,J.T.; formal analysis, J.C.; writing—review and editing,J.T.; project administration, J.T..All authors contributed to the study conception and design. All authors read and approved the final manuscript.

Funding

The authors have not disclosed any funding.

Competing Interests

The authors have no conflict of interest.

Data Availability

Data is available in the manuscript.

References

[1] Chen Jixin.Strength training in the physical fitness of youth basketball players [J].Contemporary sports technology, 2019, 9(16): 32-33.

[2] Li D, Yi C, Gu Y. Research on college physical education and sports training based on virtual reality technology[J]. Mathematical Problems in Engineering, 2021, 2021: 1-8.

[3] Wei S, Huang P, Li R, Liu Z, Zou Y. Exploring the application of artificial intelligence in sports training: a case study approach[J]. Complexity, 2021, 2021: 1-8.

[4] Zhang Y, Hou X. Application of video image processing in sports action recognition based on particle swarm optimization algorithm[J]. Preventive Medicine, 2023, 173: 107592.

[5] Jiang W. Application of motion trajectory recognition based on remote sensing image optical processing in optimizing swimming training schemes[J]. Optical and Quantum Electronics, 2024, 56(2): 264.

[6] Pickering C, Kiely J. The development of a personalised training framework: Implementation of emerging technologies for performance[J]. Journal of Functional Morphology and Kinesiology, 2019,

4(2): 25.
[7] Li G, Yang X. The Training Gesture Recognition and early warning method based on CNN Model[J]. Wireless Personal Communications, 2023: 1-16.
[8] Lei M A. Application of athletic training in the field for university students[J]. Revista Brasileira de Medicina do Esporte, 2022, 29: e2022_0219.
[9] Liu J, Wang L. The application of human–computer interaction technology fused with artificial intelligence in sports moving target detection education for college athlete[J]. Frontiers in Psychology, 2021, 12: 677590.
[10] Wang H. Research on Market Positioning and Market Development Strategy of Private Sports Training Institutions under the Condition of the Reform of the High School Entrance Examination System[J]. Highlights in Business, Economics and Management, 2022, 4: 141-147.
[11] Ghanvatkar S, Kankanhalli A, Rajan V. User models for personalized physical activity interventions: scoping review[J]. JMIR mHealth and uHealth, 2019, 7(1): e11098.
[12] Lin C C, Lin Y S, Yeh C H, Huang C C, Kuo L C, Su F C. An Exergame-Integrated IoT-Based Ergometer System Delivers Personalized Training Programs for Older Adults and Enhances Physical Fitness: A Pilot Randomized Controlled Trial[J]. Gerontology, 2023, 69(6): 768-782.
[13] Shin D, Hsieh G, Kim Y H. PlanFitting: Tailoring Personalized Exercise Plans with Large Language Models[J]. arXiv preprint arXiv:2309.12555, 2023.
[14] Cao X, Zhao X, Tang H, Fan N, Zereg F. Football players' strength training method using image processing based on machine learning[J]. Plos one, 2023, 18(6): e0287433.
[15] Li Q, Shi Q. Research on Gymnastics Teaching and Training Simulation System under Computer Internet of Things Technology[J]. Frontiers in Computing and Intelligent Systems, 2023, 6(3): 1-3.
[16] Zhou Chuang, Ju Xingguo, Li Ziang, Liu Xiaomin. Seismic initial detection based on deep convolutional GAN [J]. Petroleum geophysical exploration, 2020, 59(5): 795-803.
[17] Striuk O, Kondratenko Y. Generative adversarial neural networks and deep learning: successful cases and advanced approaches[J]. International Journal of Computing, 2021, 20(3): 339-349.
[18] Wu Xiaoguang, Deng Wenqiang, Niu Xiaochen, Jia Zheheng, Liu Shaowei. Human gait generation based on conditional GAN [J]. Journal of Instrumentation, 2020, 41(1): 129-137.
[19] Cao X, Sun G, Yu H, Guizani M. PerFED-GAN: Personalized federated learning via generative adversarial networks[J]. IEEE Internet of Things Journal, 2022, 10(5): 3749-3762.
[20] Wen J, Zhu X R, Wang C D, Tian Z. A framework for personalized recommendation with conditional generative adversarial networks[J]. Knowledge and information systems, 2022, 64(10): 2637-2660.
[21] Yoon J, Drumright L N, Van Der Schaar M. Anonymization through data synthesis using generative adversarial networks (ads-gan)[J]. IEEE journal of biomedical and health informatics, 2020, 24(8): 2378-2388.
[22] Yuan C, Huang Y. Personalized sentence generation using generative adversarial networks with author-specific word usage[J]. Computación y Sistemas, 2020, 24(1): 17-28.
[23] Ali Z, Qi G, Muhammad K, Kefalas P, Khusro S. Global citation recommendation employing generative adversarial network[J]. Expert Systems with Applications, 2021, 180: 114888.
[24] Shi Qian, Luo Ronglei. Research progress on GAN-based clothing image generation [J]. Modern textile technology, 2023, 31(2): 36.
[25] Sun Jinyang, Liu Baisong, Ren Hao, Qian Jiangbo. TAGAN: An adversarial recommendation algorithm for academic papers that combines fine-grained semantic features [J]. Telecommunications

Science, 37(8):57-65.
[26] Chen Jiwei, Wang Haitao, Jiang Ying, etc. Sequence recommendation algorithm based on generating confrontation model [J]. Chinese Journal of Informatics, 2022, 36(7): 143-153.
[27] Gao Q, Zhou F, Zhang K, Zhang F, Trajcevski G. Adversarial human trajectory learning for trip recommendation[J]. IEEE Transactions on Neural Networks and Learning Systems, 2021, 34(4): 1764-1776.
[28] Waheed W, Deng G, Liu B. Discrete Laplacian operator and its applications in signal processing[J]. IEEE Access, 2020, 8: 89692-89707.
[29] Bulut F. Low dynamic range histogram equalization (LDR-HE) via quantized Haar wavelet transform[J]. The Visual Computer, 2022, 38(6): 2239-2255.
[30] Gupta M, Taneja H, Chand L. Performance enhancement and analysis of filters in ultrasound image denoising[J]. Procedia computer science, 2018, 132: 643-652.
[31] Wang Z, Wang J, Wang Y. An intelligent diagnosis scheme based on generative adversarial learning deep neural networks and its application to planetary gearbox fault pattern recognition[J]. Neurocomputing, 2018, 310: 213-222.
[32] Kusiak A. Convolutional and generative adversarial neural networks in manufacturing[J]. International Journal of Production Research, 2020, 58(5): 1594-1604.
[33] Dash A, Ye J, Wang G. A review of Generative Adversarial Networks (GANs) and its applications in a wide variety of disciplines: From Medical to Remote Sensing[J]. IEEE Access, 2023,12: 18330-18357.
[34] Xue Z. A general generative adversarial capsule network for hyperspectral image spectral-spatial classification[J]. Remote Sensing Letters, 2020, 11(1): 19-28.
[35] Daihong J, Sai Z, Lei D, Yueming D. Multi-scale generative adversarial network for image super-resolution[J]. Soft Computing, 2022, 26(8): 3631-3641.
